# A Phase I Clinical Trial of Systemically Delivered NEMO Binding Domain Peptide in Dogs with Spontaneous Activated B-Cell like Diffuse Large B-Cell Lymphoma

**DOI:** 10.1371/journal.pone.0095404

**Published:** 2014-05-05

**Authors:** Georges Habineza Ndikuyeze, Anita Gaurnier-Hausser, Reema Patel, Albert S. Baldwin, Michael J. May, Patrick Flood, Erika Krick, Kathleen J. Propert, Nicola J. Mason

**Affiliations:** 1 Division of Hematology/Oncology, School of Medicine, University of Pennsylvania, Philadelphia, Pennsylvania, United States of America; 2 Office of Professional Studies in the Health Sciences, Drexel University College of Medicine, Philadelphia, Pennsylvania, United States of America; 3 Antech Diagnostics, New Hyde Park, New York, United States of America; 4 TheraLogics, Inc., Chapel Hill, North Carolina, United States of America; 5 Lineberger Comprehensive Cancer Center and Department of Biology, University of North Carolina, Chapel Hill, North Carolina, United States of America; 6 Department of Animal Biology, School of Veterinary Medicine, University of Pennsylvania, Philadelphia, Pennsylvania, United States of America; 7 7-020G Katz Centre for Pharmacy and Health Research, The University of Alberta, Edmonton, Alberta, Canada; 8 Department of Clinical Studies, School of Veterinary Medicine, University of Pennsylvania, Philadelphia, Pennsylvania, United States of America; 9 Department of Biostatistics and Epidemiology, University of Pennsylvania School of Medicine, Philadelphia, Pennsylvania, United States of America; 10 Department of Pathobiology, School of Veterinary Medicine, University of Pennsylvania, Philadelphia, Pennsylvania, United States of America; University of Navarra, Center for Applied Medical Research, Spain

## Abstract

Activated B-Cell (ABC) Diffuse Large B-Cell Lymphoma (DLBCL) is a common, aggressive and poorly chemoresponsive subtype of DLBCL, characterized by constitutive canonical NF-κB signaling. Inhibition of NF-κB signaling leads to apoptosis of ABC-DLBCL cell lines, suggesting targeted disruption of this pathway may have therapeutic relevance. The selective IKK inhibitor, NEMO Binding Domain (NBD) peptide effectively blocks constitutive NF-κB activity and induces apoptosis in ABC-DLBCL cells *in vitro*. Here we used a comparative approach to determine the safety and efficacy of systemic NBD peptide to inhibit constitutive NF-κB signaling in privately owned dogs with spontaneous newly diagnosed or relapsed ABC-like DLBCL. Malignant lymph nodes biopsies were taken before and twenty-four hours after peptide administration to determine biological effects. Intravenous administration of <2 mg/kg NBD peptide was safe and inhibited constitutive canonical NF-κB activity in 6/10 dogs. Reductions in mitotic index and Cyclin D expression also occurred in a subset of dogs 24 hours post peptide and in 3 dogs marked, therapeutically beneficial histopathological changes were identified. Mild, grade 1 toxicities were noted in 3 dogs at the time of peptide administration and one dog developed transient subclinical hepatopathy. Long term toxicities were not identified. Pharmacokinetic data suggested rapid uptake of peptide into tissues. No significant hematological or biochemical toxicities were identified. Overall the results from this phase I study indicate that systemic administration of NBD peptide is safe and effectively blocks constitutive NF-κB signaling and reduces malignant B cell proliferation in a subset of dogs with ABC-like DLBCL. These results have potential translational relevance for human ABC-DLBCL.

## Introduction

Diffuse large B-cell lymphoma (DLBCL) represents a heterogenous group of malignancies that can be further divided into 3 distinct sub-types: Germinal Center B Cell- (GCB-), Activated B Cell- (ABC-) and Primary Mediastinal B Cell- (PMBC-) DLBCL based on the malignant cell of origin and defined gene expression profiles [1]–[3]. ABC-DLBCL is the most aggressive and least chemo-responsive subtype with a 5 year survival rate of ∼40% [1]. ABC-DLBCL is characterized by constitutive canonical NF-κB activity that drives lymphomagenesis through up-regulation of NF-κB target genes that promote cellular proliferation (e.g. *Cyclin D2*) and survival (e.g. *XIAP, c-FLIP*, and *Bcl-2*) [4]–[8]. This mechanism contributes to the aggressive phenotype and chemo-resistance frequently recognized in patients with ABC-DLBCL.

Many molecular aberrancies responsible for constitutive NF-κB signaling in ABC-DLBCL and other hematological malignancies have been identified [9]. Amongst the most frequently occurring are oncogenic mutations in proteins of the BCR and TLR signaling pathways such as CD79a, CARD11 and MyD88, which result in constitutive IKK activity [1], [10]. ABC-DLBCL cell lines are dependent upon constitutive NF-κB signaling for their survival and disruption of this pathway using small molecules inhibitors and dominant negative IκBα constructs leads to rapid apoptosis [5], [6].

The IKK complex is central to the activation of canonical NF-κB and is composed of two catalytic subunits IKKα and IKKβ and a regulatory, ubiquitin binding subunit IKKγ or NF-κB essential modulator (NEMO) which has no catalytic function [11], [12]. IKKα and IKKβ both contain a NEMO Binding Domain (NBD) which interacts with the alpha helical domain of NEMO [13]. When activated, the IKK complex phosphorylates inhibitory IκB proteins, targeting them for ubiquitination and proteosomal degradation. This allows IκB sequestered NF-κB family members to translocate into the nucleus and initiate transcription of target genes [8]. As many of the oncogenic mutations that drive constitutive NF-κB activity in ABC-DLBCL occur upstream of the IKK complex, functional disruption of this complex represents an attractive universal strategy for the treatment of ABC-DLBCL that arises from different oncogenic mutations [14].

NBD peptide is a 13 amino acid peptide that spans the NBD of IKKα and IKKβ [13], [15]. It is a selective, non-catalytic inhibitor of IKK that disrupts the interaction of the catalytic subunits with NEMO, effectively blocking kinase activity that is triggered by many different inflammatory stimuli [15]. Interestingly, NBD peptide does not block basal NF-κB activity and following systemic administration, it safely and effectively ameliorates disease in multiple rodent models of acute and chronic inflammation [16]–[20]. NBD peptide also inhibits constitutive NF-κB activity in malignant cell lines derived from many different cancer types, including ABC-DLBCL resulting in increased sensitization to chemotherapeutic agents and increased apoptosis [21]–[26]. Given these findings, NBD peptide may represent a safe and effective therapy either alone or in combination with cytotoxic agents for patients with treatment-naïve or refractory ABC-DLBCL.

Pet dogs that develop Non-Hodgkins Lymphoma (NHL) are recognized as a clinically relevant, spontaneous large animal model of the human disease [25], [27]–[31]. Gene expression analysis of primary canine B cell lymphoma tissue has revealed germinal center and post-germinal center, ABC-like DLBCL subsets with the latter characterized by the presence of phosphorylated p65 and increased expression of NF-κB target genes [29]. Furthermore, tissue microarrays from lymphomatous canine tissue confirmed the presence of constitutive NF-κB activity and pharmacological inhibition of IKK leads to increased cytotoxicity in canine B cell lines [28]. These data support our previous findings of constitutive canonical NF-κB activity as defined by the presence of phosphorylated IKK and IκBα within the malignant lymph nodes of dogs with DLBCL [25]. Using this canine model, we have shown *in vitro* that NBD peptide, fused to the cell penetrating Antennapedia peptide (AntP) inhibits phosphorylation of IKK and IκBα in primary malignant canine B cells and leads to rapid apoptosis [25]. Furthermore, local, intranodal administration of NBD peptide to dogs with spontaneous ABC-like DLBCL effectively inhibits the expression of NF-κB target genes within the malignant node and decreases tumor burden [25]. In the present phase I clinical trial, we sought to expand on these findings and determine whether systemic, intravenous administration of NBD peptide is safe and effective in inhibiting constitutive canonical NF-κB signaling in lymphomatous tissue of dogs with spontaneous ABC-like DLBCL.

## Materials and Methods

### Reagents and cell lines

NBD peptide was synthesized and purified by Dr. James I. Elliott (at the Howard Hughes Medical Institute Biopolymer-Keck Foundation Biotechnology Resource Laboratory, Yale University, New Haven, CT) using standard tertbutoxycarbonyl (t-Boc) chemistry, cleavage with hydrofluoric acid, and purification by reversed-phase HPLC [26]. TNFα was purchased from Sigma-Aldrich (Saint Louis, MO). The human GCB-DLBCL cell line SUDHL-6 and the ABC-DLBCL cell line OCI-Ly10 were obtained from Dr Anne Novak (Mayo Clinic Cancer Center, Rochester, Mn).

### Clinical trial study population, eligibility criteria and study design

The clinical trial protocol was fully approved by the University of Pennsylvania's Institutional Animal Care and Use Committee (Privately Owned Animal Protocol number: 803765). Only dogs with full written informed owner consent were eligible for enrollment. Pet dogs with newly diagnosed (treatment-naïve) or relapsed stage III or greater, lymphoma were eligible for screening if they were systemically well (as determined by clinical examination, blood screening (complete blood counts (CBC) and Chemistry Screen (CS) and urinalysis (UA)), had a PCV>30%, an ECOG performance <1, a life expectancy of >2 months and had not received systemic chemotherapy within the last 3 weeks or prednisone therapy within the last 72 hours. All dogs underwent lymph node excision or biopsy to confirm the diagnosis of DLBCL by histopathology and flow cytometric phenotyping and to determine the presence of constitutive NF-κB activity. The histopathological diagnosis of DLBCL and cytological confirmation of large B cell lymphoma were made by board certified veterinary pathologists (M.G., and A.D., see acknowledgements) and by a board certified clinical pathologist (R.P.) respectively. Only dogs with DLBCL and constitutive NF-κB activity within their malignant lymph nodes as determined by the presence of phospho-IKK, phospho-IκBα and phospho-p65 were eligible to receive NBD peptide.

Tumor burden was calculated by measuring three dimensions (length, width and height) for each malignant lymph node. The mass of each node was calculated as previously described and the sum of measured lymph node mass was considered as total tumor mass [25]. Response to NBD peptide was evaluated according to the Veterinary Cooperative Oncology Group criteria for assessment of response in peripheral nodal lymphoma, adapted for use in dogs from the published RECIST criteria [32]. The Veterinary Cooperative Oncology Group Common Terminology for Adverse Events, version 1.0 was used to grade adverse events [33].

### In vivo NBD peptide treatment

In this phase I dose escalation clinical trial, both treatment-naïve and relapsed dogs were enrolled. NBD peptide was administered according to a standard 3+3 trial design. The dose endpoint was the dose at which canonical NF-κB signaling was inhibited within malignant lymph nodes. Peptide was reconstituted to 50 mM in sterile DMSO and diluted in 20 mls of lactated Ringers solution immediately prior to intravenous administration at a constant rate of 20 ml/hr. Twenty-four hours post NBD peptide administration, excisional or Tru-cut biopsies of a peripheral malignant lymph node were taken for Western blot, qRT-PCR and histopathological analysis. Following the post-treatment biopsy, dogs received induction or rescue chemotherapy as determined by their primary attending veterinary oncologist and a short course of prophylactic antibiotics (amoxicillin). All dogs received a follow-up full clinical examination, CBC, CS and urinalysis one week post NBD peptide.

### Pharmacokinetics

EDTA anti-coagulated blood samples were taken at 0, 30, 60, 90, 120, 240, 360 mins and 24 hours post infusion. Plasma was separated by centrifugation and stored at −80°C prior to pharmacokinetic analysis. Mass spectrometer analysis of NBD peptide in plasma samples was performed by Frontage Laboratories, Exton, PA and pharmacokinetic (PK) parameters were determined using WinNonlin v5.2 software. The lower limit of quantification (LLOQ) was set to 10 ng/ml however, in some instances the LLOQ was changed to 20 ng/ml because of potential interference in the samples.

### Flow cytometry

Lymphomas were phenotyped using a combination of the following antibodies: Fluorescein isothiocyanate (FITC)-conjugated rat anti-canine CD3 (Serotec, Raleigh NC), Phycoerythrin (PE)-conjugated mouse anti-canine CD21-like molecule (Serotec), allophycocyanin (APC)-conjugated mouse anti-human CD79a (BD Biosciences, San Jose, CA), and APC-conjugated mouse anti-canine CD45. For CD79a staining, cells were fixed with 1% paraformaldehyde and permeabilized with 0.1% saponin before staining. Cells were acquired on a FACSCalibur cytometer (BD Biosciences) and analyzed using FlowJo software (TreeStar).

### Western blot analyses

Lymph node biopsy samples were mechanically dissociated in RPMI and red blood cells were removed using ACK lysis buffer (Gibco) according to manufacturer's instructions. Cells were lysed in 50 mM Tris-HCL containing 1% NP-40, 150 mM NaCl, 2.5 mM EDTA, 5% glycerol, a 1∶50 dilution of Protease Inhibitor Cocktail and 1∶50 dilutions of Phosphatase Inhibitor Cocktails 2 and 3 (Sigma-Aldrich, Saint Louis, MO). Samples were centrifuged and protein concentrations were determined by micro BCA assay (Thermo Scientific, Rockford, IL). Proteins from whole cell extracts were separated by SDS-PAGE (10%) followed by transfer to PVDF membrane. Membranes were probed with polyclonal rabbit anti-human antibodies against phospho-IKKα/β (16A6), IKKβ (L570), phospho-IκBα (14D4), mouse anti-human IκBα (L35A5) and β-actin (4967) (Cell Signaling, Danvers, MA). An HRP-conjugated donkey anti-rabbit or anti-mouse IgG was used as the secondary detection antibody (Amersham, Piscataway, NJ). Blots were developed using ECL Plus (Amersham, Piscataway, NJ). Densitometry was performed using ImageJ software and integrated density values for each band were obtained using the Analyze Particles function [34].

### Quantitative Reverse Transcription PCR (qRT-PCR)

Lymph node biopsies were immediately placed in RNAlater (Ambion, Austin, TX) and stored at –80°C prior to RNA extraction. Total RNA extraction was performed using the RNeasy Mini Kit (Qiagen, Valencia, CA). Reverse transcription was performed using random hexamers and Superscript II reverse transcriptase (Invitrogen Corp., Carlsbad. CA, USA) according to the manufacturer's instructions. Primer sequences for canine A1, A20, Bcl-XL, c-FLIP, Cyclin D1, Bcl-2, XIAP and β-actin have been previously described [25], [35]. Primers were synthesized by Invitrogen Corp. Real-time PCR assays were performed using SYBR Green (Fermentas, Glen Burnie, Maryland). Samples were run in triplicate using standard conditions on an ABI 7500 sequence detector (Applied Biosystems, Carlsbad, CA) and data were analyzed using β-actin as an endogenous control. Dissociation curves were performed after each experiment to confirm the specificity of product amplification. Statistical analysis was performed using a two-tailed Student's ‘t’ test where statistical significance  = p<0.05.

### Immunohistochemistry (Ki67, IRF4 and TUNEL staining)

Immunohistochemical analysis was performed on 5 micron sections of formalin fixed paraffin embedded lymph node tissue from dogs with spontaneous DLBCL using a DAKO automatic universal staining system. Sections were heated in a 60°C oven for 1 hour, depararaffinized in PRO PAR clearant and rehydrated in progressively decreasing grades of ethanol. Antigen retrieval was performed by boiling sections in sodium citrate buffer, pH ∼9.0 for 15 minutes. Endogenous peroxidase was blocked with 3% hydrogen peroxide. For Ki67 staining, a 1∶50 dilution of a monoclonal mouse anti-human antibody (Clone MIB-1, DAKO) was used and immunodetection was performed using a biotinylated goat anti-rabbit/anti-mouse immunoglobulin and SA-HRP (LSAB2 kit, DAKO, Carpinteria, CA). For IRF-4 staining, a 1∶200 dilution of a polyclonal mouse anti human antibody (DAKO, M7259) was used and immunodetection was performed using a peroxidase polymer conjugated anti-mouse secondary antibody system (ImmPRESS Anti-mouse Ig peroxidase polymer detection kit). In all cases, antibody binding was detected using 3,3′-diaminobenzidine solution and tissues were counterstained with hematoxylin. Slides were viewed using a Nikon E600 infinity corrected upright microscope. Bright field images were acquired using a Nikon Digital Sight DS-Fi1 color camera using NIS-Element BR3.0 for image analysis. For TUNEL staining, formalin fixed, paraffin embedded tissue sections were de-waxed, rehydrated and permeabilized in 10 mM sodium citrate buffer. Apoptotic cells were detected using the cell death detection kit (Roche, Indianapolis, IA) following the manufacturer's instructions. Nuclei were counterstained with DAPI, and slides were mounted with ProLong Antifade (Invitrogen) prior to visualization.

## Results

### Constitutive canonical NF-κB activity is present in dogs with B cell lymphoma

Eleven dogs with spontaneous DLBCL that fulfilled all eligibility criteria were enrolled following owner consent in this phase I pre-clinical feasibility trial. Patient characteristics are shown in Table S1. At the time of presentation, all dogs were diagnosed with stage III or greater, substage a, lymphoma. Using both histopathology and flow cytometry, all dogs were confirmed to have intermediate to high grade DLBCL, except for one dog where insufficient tissue was obtained to allow histopathological classification. This case was confirmed as having large B cell lymphoma by flow cytometry and cytological analysis. In all cases evaluated by histopathology the normal lymph node architecture was destroyed by sheets of neoplastic lymphocytes and the number of mitoses ranged from 3–7 per high power field (intermediate grade) or 8–12 per high power field (high grade) (Table S1).

**Figure 1:**
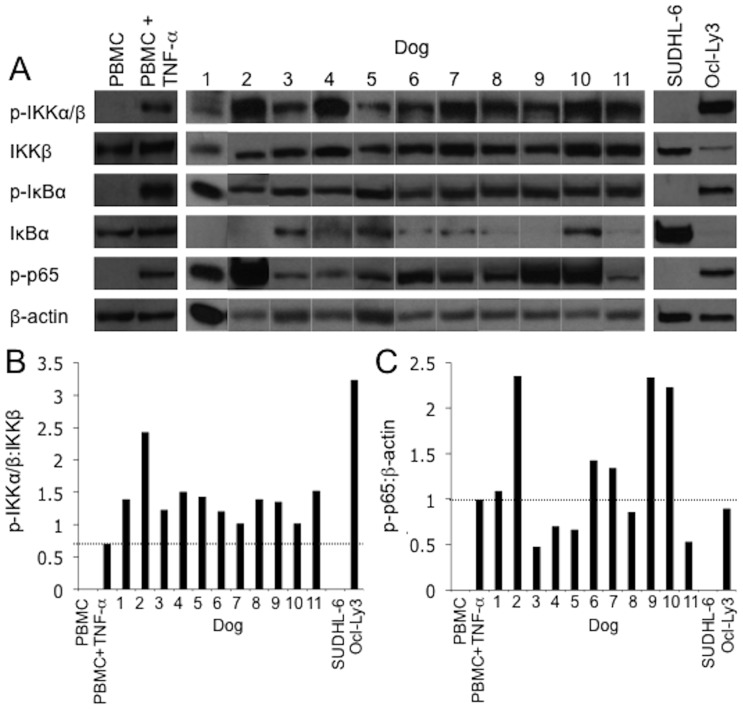
Constitutive canonical NF-κB activity is present in dogs with large B cell lymphoma. **A**. Whole cell extracts from malignant lymph node biopsies were analyzed by immunoblot for the presence of phospho-IKKα/β, IKKβ, phospho-IκBα, IκBα, phospho-p65 and β-actin. WCE from TNF-α activated canine PBMCs and from the human ABC-DLBCL cell line Ocl-Ly3 were used as positive controls and WCE from unstimulated canine PBMCs and the GCB-DLBCL cell line SUDHL-6 were used as negative controls. Integrated densitometry was used to quantify amounts of phospho-IKKα/β and IKKβ and phospho-p65 and β-actin. Relative canonical NF-κB activity in each sample was expressed as **B**. phospho-IKKα/β:IKKβ and **C**. phospho-p65:β-actin. The horizontal line in **B** and **C** marks the phosphorylation status of IKKα/β and phospho-p65 respectively in TNF-α activated canine PBMCs.

To confirm the presence of constitutive canonical NF-κB activity within malignant lymph nodes, Western blot was performed on whole cell extracts prepared from malignant peripheral lymph node biopsies. All dogs screened had evidence of p-IKK, p-IκBα and p-p65 in their malignant nodes (Fig. 1A). These findings were consistent with those of the human ABC-DLBCL cell line Ocl-Ly3 which exhibits constitutive NF-κB activity [5] and of TNF-α activated canine PBMCs (positive canine control) (Fig. 1A). In contrast, in the human GCB-DLBCL cell line SUDHL-6 which does not exhibit constitutive NF-κB activity [5] and resting canine PBMCs, p-IKK, p-IκBα and p-p65 were not detected (Fig. 1A). Densitometry was performed to quantify phosphorylation of IKK and p65 (Fig. 1B and C). All dogs showed p-IKK:IKK ratios similar to or greater than TNF-α activated PBMCs. Although no dog exhibited pIKK:IKK ratios greater than Ocl-Ly3, seven out of eleven dogs showed greater or equal p-p65:β-actin ratios compared to Ocl-Ly3. Interestingly, although all eligible dogs demonstrated evidence of constitutive canonical NF-κB activity in malignant lymph node biopsies, suggesting an ABC phenotype, none of the malignant canine lymph nodes stained positive for MUM1/IRF-4, an NF-κB target gene that is over-expressed in ABC-DLBCL and a hallmark of this subtype in humans (data not shown). Based on these results, all enrolled dogs were considered to have “ABC-like” DLBCL.

### Systemic administration of NBD peptide is safe and has minimal effects on hematological and biochemical parameters

In this phase I, 3+3 standard dose escalation study, three dogs in Group 1 received 0.5 mg/kg NBD peptide, seven dogs in Group 2 received 1 mg/kg NBD peptide and one dog in Group 3 received 2 mg/kg NBD peptide. NBD peptide was administered as a slow intravenous infusion over 1 hour. During infusion, two dogs in Group 1 became drowsy and developed moderate tachycardia and hypertension (Table S1). These effects were mild and transient and resolved without treatment when the infusion was discontinued. The remaining dogs were premedicated with the serotonin 5-HT3 receptor antagonist ondansetron and the H1 receptor antagonist diphenhydramine. Transient hypertension was identified in one additional dog in Group 2. Pharmacokinetic analysis showed detectable albeit low levels of NBD peptide in plasma samples of 8/10 dogs within 60 minutes of infusion (Table S2). Peptide was not detected in the plasma of any dog after 60 mins.

Hematological and biochemical parameters were evaluated for all dogs immediately before and one week after NBD peptide plus induction or rescue chemotherapy (Fig. S1). There were mild decreases in hematocrit (9/11 dogs) and lymphocyte counts (9/11 dogs) and an increase in platelet count (8/11 dogs) post combination treatment. However, in most cases post treatment values remained within the normal range. In dogs with elevated ALKP values prior to NBD peptide administration (4/11 dogs; all previously treated for relapsed lymphoma), further increases in ALKP were seen post treatment. One dog in Group 2 with normal serum biochemical parameters at the time of treatment and who received L'asparaginase 24 hrs after peptide administration, showed significant but asymptomatic increases in liver values (ALT, ALKP, AST and GGT) one week later (Fig. S1). The dog received no specific treatment and liver values returned to baseline two weeks later (data not shown). For this reason, 3 more dogs were recruited to Group 2 before further dose escalation. No other dog showed significant biochemical abnormalities following treatment.

### NBD peptide inhibits constitutive canonical NF-κB signaling in malignant lymph nodes

**Figure 2:**
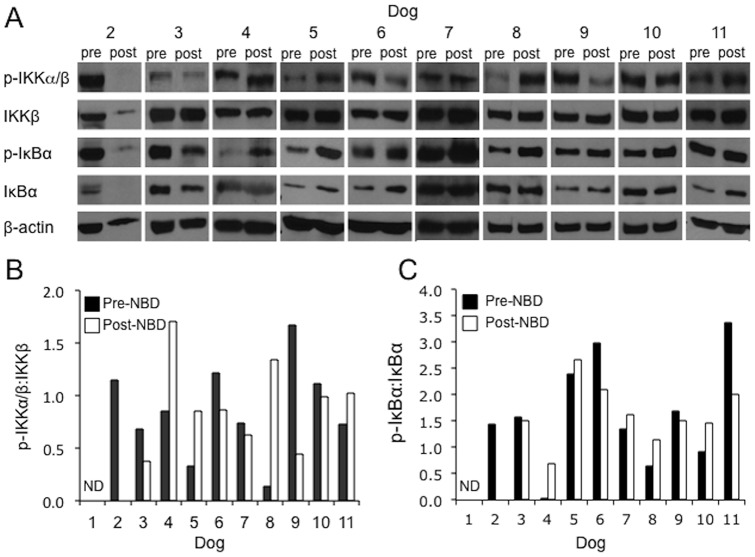
Systemic administration of NBD peptide inhibits IKK and IκBα phosphorylation in malignant lymphoid tissue. Biopsies of malignant lymph nodes taken before (pre-) and 24 hours after (post-) NBD peptide administration were evaluated for the presence of phospho-IKKα/β and phospho-IκBα by immunoblot. **A**. Immunoblots for phospho-IKKα/β and IKKβ and phospho-IκBα and IκBα. Immunoblots were evaluated by densitometry and the ratios of **B**. phospho-IKKα/β:IKKβ and **C**. phospho-IκBα:IκBα were calculated. ND (Not Determined).

To determine whether NBD peptide can inhibit constitutive canonical NF-κB signaling *in vivo*, biopsies of malignant lymph nodes were taken before and 24 hours after intravenous peptide administration, prior to induction or rescue chemotherapy. Previous mouse studies have demonstrated inhibitory effects of NBD peptide on NF-κB activity occurring between 24 and 48 hours after systemic administration [17], [18], [36]. The effects of NBD peptide on the phosphorylation status of IKK and IκBα were determined by immunoblot (Fig. 2A). To correct for any unequal sample loading, ratios of pIKK:IKK and pIκBα:IκBα were determined in pre- and post-peptide biopsy samples using integrated densitometry. Paired samples from ten of the eleven treated dogs were available for analysis (Fig. 2A). Six out of ten dogs showed reductions in the phosphorylation status of IKK (2 in Group 1 and 4 in Group 2) and 4 dogs showed an increase in IKK phosphorylation (3 in Group 2 and 1 in Group 3) 24 hours post peptide. Changes in phosphorylation status of IκBα generally reflected the observed change in IKK phosphorylation (Fig. 2C). Bands for p-IKK and IκBα were not detectable for the post treatment sample of dog 2, however p-IκBα:β-actin ratio was reduced in the post sample compared to pre-sample (data not shown). In all other dogs, the effects of NBD peptide on p-IKK:β-actin and p-IκBα:β-actin correlated well with effects on pIKK:IKK and pIκBα:IκBα (data not shown).

Based on evidence of biological activity of NBD peptide when administered at 1 mg/kg, no clear dose dependent effect on inhibition at 2 mg/kg, and the significant cost of peptide administration at doses greater than 1 mg/kg no further dose escalation was performed.

### NBD peptide inhibits proliferation and induces apoptosis in a subset of dogs with spontaneous DLBCL

**Figure 3:**
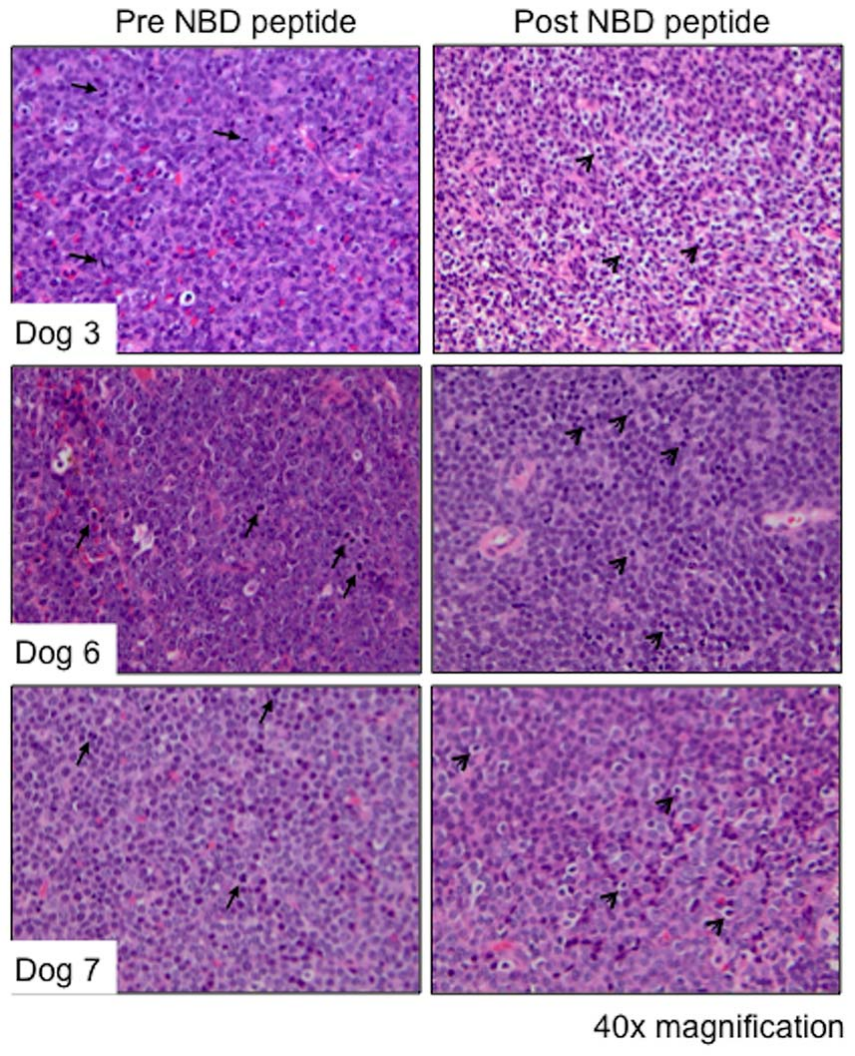
Effect of NBD peptide on malignant lymph node histopathology. Biopsies of malignant lymph nodes taken before (pre-) and 24 hours after (post-) NBD peptide adminsitration were stained with H&E and evaluated by light microscopy. Sections were evaluated for the presence of mitotic figures, cellular shrinkage, nuclear pyknosis, apoptotic bodies and tingible body macrophages. Arrows indicate mitotic figures, arrowheads indicate pyknotic nuclei and cellular shrinkage. Representative sections of three dogs are shown (40× magnification).

**Figure 4:**
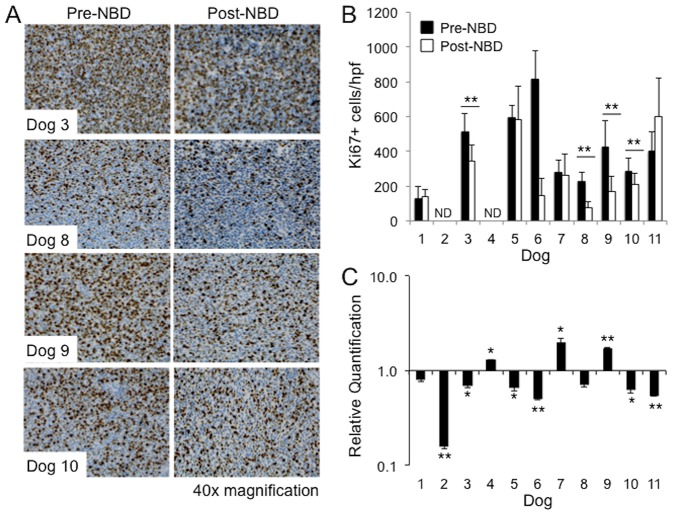
NBD peptide inhibits malignant cell proliferation and Cyclin D expression in a subset of dogs with ABC-like DLBCL **A.** Malignant lymph node tissue sections taken before (pre-) and 24 hours after (post-) NBD peptide treatment were evaluated for expression of Ki67 by IHC. **B**. 10 high power fields were evaluated for Ki67 positive cells in each pre- and post-NBD peptide tissue section. The average number of Ki67 positive cells per high power field (hpf) was determined. **C**. Quantitative RT-PCR analysis of CyclinD expression in malignant tumor samples post-NBD peptide. The relative quantification of gene expression in post treatment samples was normalized to pre-treatment values for each dog. All assays were performed in triplicate and β-actin was used as an endogenous control. ^★^p<0.05, ^★★^p<0.005, ND (Not Determined).

Constitutive canonical NF-κB signaling contributes to lymphomagenesis by driving cellular proliferation and inhibiting apoptosis. Therefore to determine the effects of NBD peptide on cellular proliferation and apoptosis the histopathological features of lymph node biopsies taken pre and 24 hours post peptide treatment were compared. Paired tissue samples were available for histopathological analysis from nine of the eleven dogs. In 3 dogs, post treatment samples had notably fewer mitotic figures, increased numbers of apoptotic bodies and tingible body macrophages and exhibited malignant cell shrinkage, nuclear pyknosis and fragmentation when compared to pre-treatment biopsies (Fig. 3). To quantify the effect of NBD peptide on malignant cell proliferation, pre- and 24 hours post-peptide treatment biopsy samples were evaluated for expression of the proliferation marker Ki67 by IHC (Fig. 4A and B). Four dogs showed a statistically significant reduction in the number of Ki67^+^ cells following NBD peptide treatment. Dog 6 also showed a reduction in mitotic index however the post treatment biopsy sample was too small to evaluate 10 high-powered fields and so statistical significance could not be determined. To determine whether NBD peptide inhibited the expression of *Cyclin D1*, qRT-PCR was performed on pre- and 24 hour post-peptide biopsy samples. Eight out of eleven dogs showed a decrease in the expression of *Cyclin D1* following NBD peptide treatment (Fig. 4C) and 4 of these dogs also had a reduction in Ki67 mitotic index.

### Effects of NBD peptide on cellular apoptosis and tumor burden

**Figure 5:**
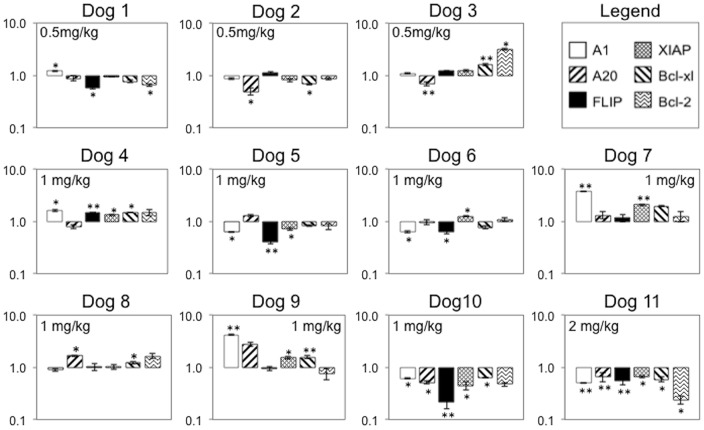
NBD inhibits the expression of canonical NF-κB target genes in a subset of dogs with ABC-like DLBCL. NF-κB target gene expression within malignant lymph node tissues before and 24 hours after NBD peptide administration was determined by qRT-PCR. The relative quantification of gene expression in post treatment samples was normalized to pre-treatment values for each dog. β-actin was used as an endogenous control and all assays were performed in triplicate. p values are calculated on dCt data, ^★^p<0.05, ^★★^p<0.005.

**Figure 6:**
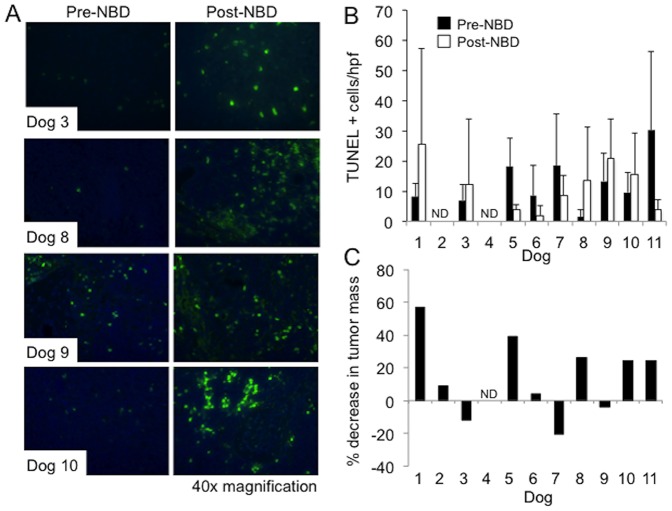
NBD peptide promotes apoptosis and reduces tumor burden in a subset of dogs with ABC-like DLBCL. **A**. Malignant lymph node tissue sections taken before (pre-) and 24 hours after (post-) NBD peptide treatment were stained using TUNEL and counterstained with DAPI. **B**. Apototic cells were counted in 10 high power fields and the average number of positive cells was determined for both pre- and post-treatment sections. **C**. Calculated percent decrease in tumor mass 24 hours after NBD peptide treatment. Measureable tumor burden was determined pre and 24 hours post NBD peptide treatment and the percentage change in tumor burden was calculated. ND (Not Determined).

The expression of six pro-survival NF-κB target genes (*A1, c-FLIP, XIAP, Bcl-XL, Bcl-2* and *A20*) were evaluated by qRT-PCR in malignant lymph nodes before and 24 hours after NBD peptide administration (Fig. 5). In 6 out of 11 dogs, the expression of 3 or more target genes was reduced following peptide administration. Although there was no apparent dose-dependent effect of NBD peptide on other measured parameters in the one dog that received 2 mg/kg NBD peptide (dog 11), it was noted that this dog showed the most significant inhibition of NF-κB target genes. To determine whether NBD peptide treatment increased apoptosis, TUNEL staining was performed on pre- and post-peptide biopsy samples (Fig 6A). Tissue was available from 9 dogs. Five dogs showed a trend towards an increase in apoptosis following treatment (Fig 6B). Although there was no clear correlation between inhibition of pro-survival gene expression and apoptosis, 4 of the dogs that showed an increase in apoptosis also showed statistically significant reductions in Ki67 index (Fig. 4B). There was no correlation between NBD peptide dose and apoptosis.

To determine whether NBD peptide reduced tumor burden, malignant lymph nodes were measured immediately before and 24 hours after NBD peptide administration. Three dimensions were measured for each node and the node mass was calculated as previously described [25]. The sum of peripheral lymph node mass was considered as measureable tumor mass. The same lymph nodes were evaluated 24 hours post NBD peptide and the change in measureable tumor mass was calculated (Fig. 6C). Measurements were available for 10 treated dogs. Five out of 10 dogs showed greater than 20% reduction in measureable tumor mass following peptide administration. Seven dogs in this phase I study achieved clinical remission (CR) following NBD peptide plus chemotherapy including 5 dogs treated at the time of their first relapse. All dogs that achieved CR showed evidence of either p-IKK inhibition or *Cyclin D1* inhibition (Table S3). The four dogs that did not achieve clinical remission experienced either poor response to initial chemotherapy or were presenting at the time of second or third relapse and were multi-drug resistant. While NBD peptide treatment lead to Ki67 and Cyclin D inhibition in three of these dogs, a single dose of NBD peptide was insufficient to induce CR.

## Discussion

Many of the genetic mutations that drive lymphomagenesis via constitutive NF-κB activation in ABC-DLBCL occur upstream of the canonical heterotrimeric IKK complex containing IKKα, IKKβ and NEMO. Therefore, specifically targeting this complex may provide therapeutic benefit for patients with ABC-DLBCL regardless of the specific mutation that disrupts signal regulation. Evaluation of small molecule ATP-binding site inhibitors of IKKβ re-inforces this concept although off-target side effects together with the compensatory activity of IKKα homodimers observed in the presence of IKKβ inhibition may reduce their therapeutic efficacy [37], [38]. In contrast, NBD peptide binds to and inhibits association of NEMO with IKKα and IKKβ making its inhibitory effect specific for the IKK complex.

In this study we recruited dogs with evidence of constitutive canonical NF-κB activity in their malignant lymph nodes as determined by the presence of phosphorylated IKK, IκBα and p65. While all dogs screened had evidence of canonical NF-κB activity according to these criteria, suggesting an ABC-like phenotype, none of the canine tumors expressed MUM-1/IRF-4, a hallmark feature of ABC-DLBCL in humans [2], [5]. In human ABC-DLBCL over-expression of IRF-4 is driven by constitutive NF-κB activity and operates in a feedback loop to reinforce constitutive NF-κB signaling. Our results therefore suggest that the downstream effectors of NF-κB activity that drive lymphomagenesis may differ between species. These findings and conclusions are supported by recent gene expression profiling studies of canine B cell lymphomas [29]. Interestingly, data from human DLBCL cell lines indicates that nuclear p65 and cytoplasmic p-IκBα are also present in GCB cell lines and that this may result from constitutive activity of the non-canonical pathway leading to stabilization of NIK [39]. Constitutive activation of both canonical and non canonical NF-κB pathways has recently been demonstrated in canine lymphoma although the mechanisms leading to this activity are as yet undetermined [28].

The aim of this study was to determine the safety and biologically effective dose of systemically administered NBD peptide in dogs with ABC-like DLBCL. Our results show that there were no significant acute or chronic toxicities identified at any NBD peptide dose evaluated (0.5–2.0 mg/kg) either by clinical or hematological and biochemical parameters and all dogs survived for at least one month after treatment. One dog did develop transient biochemical abnormalities that were consistant with hepatotoxic damage. This dog also received L′asparaginase which, although not reported in dogs, causes hepatocellular enzyme elevations and subclinical hepatopathy in humans. In all dogs, mild reductions in lymphocyte counts were detected one week after NBD peptide plus chemotherapy. We have previously shown that NBD peptide has minimal effect on peripheral B cells and consider it likely that these changes are associated with concurrent chemotherapy rather than peptide-induced B or T cell apoptosis [25]. While this safety data relates only to a single dose of peptide, cummulative toxicities following repeat dosing are unlikely to occur given the short half life of the peptide and the available safety data on multi-dose administration in rodents. Following peptide administration and chemotherapy 7 dogs achieved durable clinical remissions and three of these dogs survived greater than 16 months (Table S1). Two relapsed dogs (dogs 4 and 6) were treated with the same chemotherapeutic agents following NBD peptide as they had been resistant to before peptide administration. In these cases, the single dose of NBD peptide did not appear to reverse chemoresistance and induce a CR. However, it is likely that NBD peptide will need to be administered more than once to reverse resistance and lead to an improved clinical response. Further placebo controlled clinical trials will be required to determine whether NF-κB inhibition synergizes with chemotherapy to prolong time to progression and overall survival in dogs with DLBCL [28].

*In vivo* studies in rats, mice and piglets have evaluated NBD peptide at doses ranging from 0.75 mg/kg to 20 mg/kg and have demonstrated that following intraperitoneal, intranasal, intracranial and topical administration NBD peptide has therapeutic effects at distant sites [15], [17]–[19], [40]–[44]. In all instances, systemic administration of NBD peptide was well tolerated and showed no cytotoxic effects in the majority of tissues analysed [45], [46]. These findings suggest that the drug has good absorption and tissue distribution kinetics. In this study, as a consequence of drug expense and evidence of biological activity at 0.5–2.0 mg/kg, the maximum tolerated dose of NBD peptide in dogs was not reached and it is possible that higher doses of peptide would result in greater NF-κB inhibition. Pharmacokinetic data obtained during this study indicate that at the doses studied plasma concentrations of NBD peptide were very low and in all cases undetectable 1 hour post administration. Given that effective inhibition of canonial NF-κB activity occurred in some dogs, it is most likely that the observed plasma clearance is associated with rapid tissue uptake of peptide rather than rapid elimination.

We found that 24 hours after systemic administration, low doses (<2.0 mg/kg) of NBD peptide inhibited IKK phosphorylation and decreased malignant cell proliferation in dogs with the highest ratios of p-p65:β-actin at enrollment (Table S3). There was no apparent dose effect. Similar results were obtained when NBD peptide was administered via direct intranodal injection into malignant canine lymph nodes [25]. Five of the dogs with high ratios of p-p65:β-actin at enrollment achieved a complete remission following NBD peptide and chemotherapy. These results suggest that NBD peptide is effective in inhibiting canonical NF-κB activity in dogs with DLBCL at doses ranging from 0.5–2.0 mg/kg and that dogs whose tumors have high levels of NF-κB activity (as determined by high p-p65:β-actin) are most likely to respond [47]. Different subsets of ABC-DLBCL have been distinguished in humans based on their expression of STAT3 and the degree of NF-κB activity as determined by gene expression profiling [47]. It is possible that similar subsets of ABC-like DLBCL occur spontaneously in dogs and that this influences their response to IKK inhibitors. In the absence of definining, subtype-specific phenotypic markers further gene expression analysis would be required to determine whether variations in response to NBD peptide are truly associated with different ABC-DLBCL subtypes. Indeed, future studies will be required to accurately detect those patients (both human and canine) that exhibit high levels of NF-κB activity within their tumors and determine whether this correlates with biological and clinical response to IKK inhibitors such as NBD peptide. In cases with high levels of NF-κB activity, higher doses of NBD peptide or the concurrent use of synergistic pathway inhibitors, such as Jak/STAT inhibitors may provide greater levels of inhibition and therapeutic benefit.

Interestingly, one dog that did not have a high ratio of p-p65:β-actin at enrollment (dog 3), also showed biological effects of NBD peptide within malignant tissue including decreased IKK phosphorylation, decreased Ki67 index, inhibition of *Cyclin D1* expression, increased TUNEL staining and clear histopathological changes consistant with increased cell death. This result would be consistant with NBD mediated inhibition of an IKK dependent, NF-κB independent pathway that is important for cell survival [28]. Unexpectedly, the four dogs with the lowest ratios of p-p65:β-actin at enrollment showed an increase in p-IKK:IKK ratios following peptide treatment. It is possible that this may be due to the previously reported small transient increase in IKK activity observed in resting cells following NBD treatment [15]. However, consistent with the overall inhibition of NF-κB signaling by NBD peptide, increased p-IKK levels did not negatively effect clinical outcome as three of these dogs showed a reduction in tumor volume and two experienced clinical remission following combination peptide and chemotherapy.

In summary, NBD peptide delivered intravenously to dogs is safe and effective at inhibiting constitutive canonical NF-κB activity in a subset of dogs that exhibit the highest p-p65:β-actin within their tumors. Futher studies are required to reliably identify dogs with ABC-DLBCL that are likely to respond to NF-κB inhibition and to determine whether repeat doses of NBD peptide, administered in combination with systemic chemotherapy to dogs with relapsed, refractory ABC-like DLBCL will have substantial therapeutic benefit. The results of these studies may have important translational relevance to human patients with ABC-DLBCL.

## Supporting Information

Figure S1 Changes in hematological and biochemical parameters following NBD peptide administration. Hematology and serum biochemistry were performed before (Pre-) and one week after (Post-) NBD peptide administration. The bracket depicts reference range values for each parameter. Dashed lines represent dogs in Group 1, solid lines represent dogs in Group 2 and the dotted line represents the one dog in Group 3.
(TIF)

Table S1 Signalment, disease characteristics and outcome of clinical trial patients.
(TIF)

Table S2 Pharmacokinetic analysis of NBD peptide following intravenous administration. ND = Not Determined.
(TIF)

Table S3 Summary of effects of NBD peptide on treated dogs. X indicates dogs that showed the changes listed in the first column.
(TIF)
