# Supplementary figures and images for: A Phase I Clinical Trial of Systemically Delivered NEMO Binding Domain Peptide in Dogs with Spontaneous Activated B-Cell like Diffuse Large B-Cell Lymphoma
Source: PLoS One. 2014 May 5;9(5):e95404. doi: 10.1371/journal.pone.0095404 (PMC4010398; doi:10.1371/journal.pone.0095404)

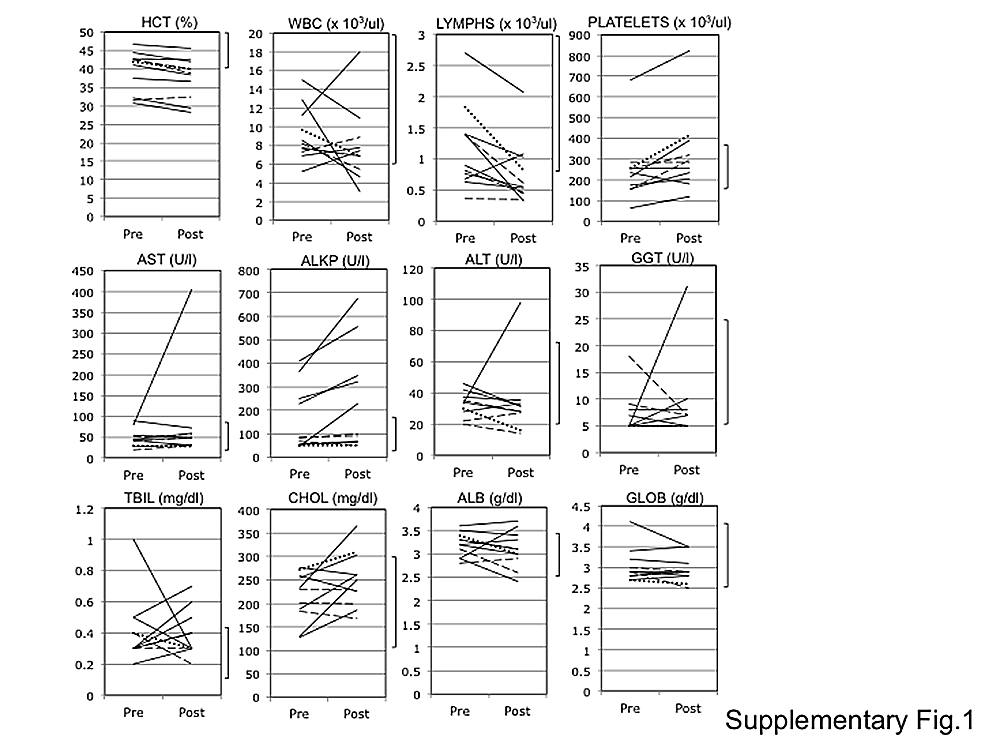

Supplement: Figure S1 — Changes in hematological and biochemical parameters following NBD peptide administration. Hematology and serum biochemistry were performed before (Pre-) and one week after (Post-) NBD peptide administration. The bracket depicts reference range values for each parameter. Dashed lines represent dogs in Group 1, solid lines represent dogs in Group 2 and the dotted line represents the one dog in Group 3. (TIF) [file pone.0095404.s001.tif]

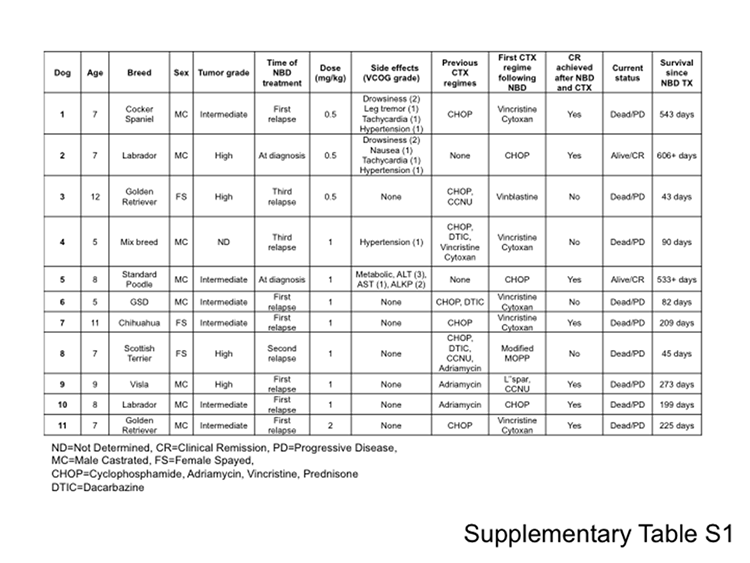

Supplement: Table S1 — Signalment, disease characteristics and outcome of clinical trial patients. (TIF) [file pone.0095404.s002.tif]

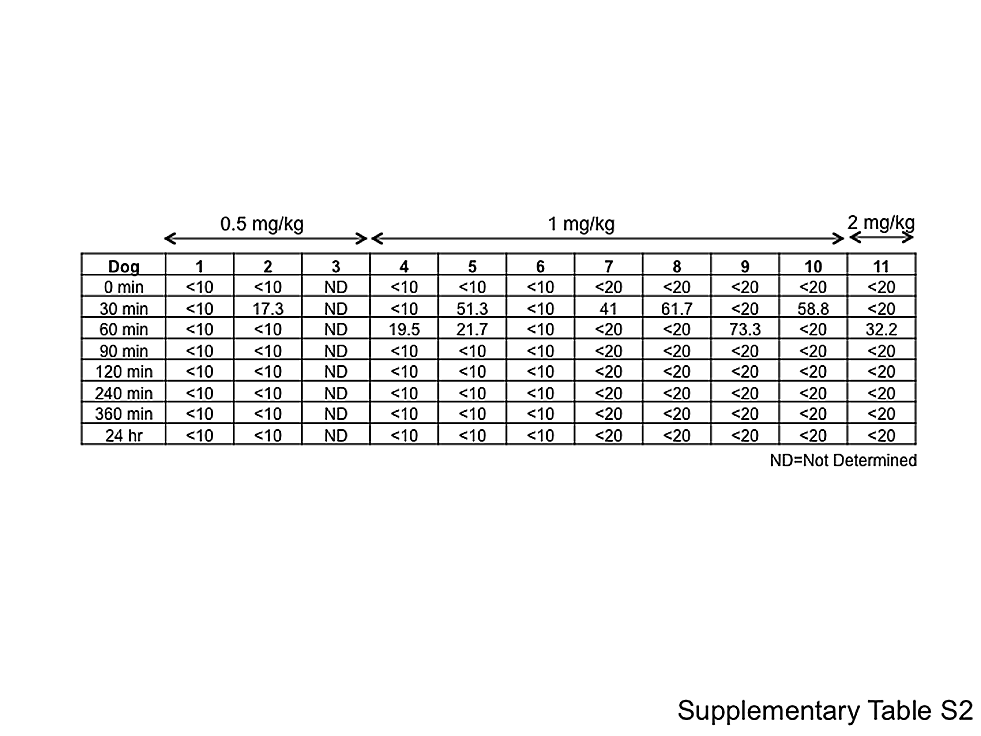

Supplement: Table S2 — Pharmacokinetic analysis of NBD peptide following intravenous administration. ND = Not Determined. (TIF) [file pone.0095404.s003.tif]

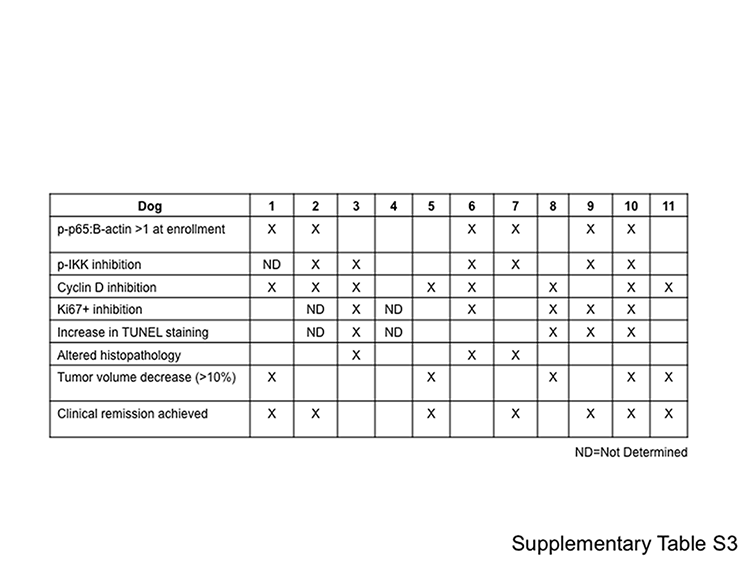

Supplement: Table S3 — Summary of effects of NBD peptide on treated dogs. X indicates dogs that showed the changes listed in the first column. (TIF) [file pone.0095404.s004.tif]
